# Effect of Restrictions on Television Food Advertising to Children on Exposure to Advertisements for ‘Less Healthy’ Foods: Repeat Cross-Sectional Study

**DOI:** 10.1371/journal.pone.0031578

**Published:** 2012-02-15

**Authors:** Jean Adams, Rachel Tyrrell, Ashley J. Adamson, Martin White

**Affiliations:** 1 Institute of Health and Society, Newcastle University, Newcastle upon Tyne, United Kingdom; 2 Human Nutrition Research Centre, Newcastle University, Newcastle upon Tyne, United Kingdom; Wayne State University School of Medicine, United States of America

## Abstract

**Background:**

In 2007, new scheduling restrictions on television food advertising to children in the UK were announced. The aim of the restrictions was to “reduce significantly the exposure of children under 16 to high fat, salt or sugar (HFSS) advertising”. We explored the impact of the restrictions on relative exposure to HFSS food advertising among all viewers and among child television viewers, as well as adherence to the restrictions.

**Methods:**

We conducted two cross-sectional studies of all advertisements broadcast in one region of the UK over one week periods – the first (week 1) six months before the restrictions were introduced, and the second (week 2) six months after. Data on what products were advertised were linked to data on how many people watched each advertisement. Nutritional content of foods advertised was added to the dataset and used to calculate HFSS status. Relative exposure was calculated as the proportion of all advertising person-minute-views (PMVs) that were for HFSS foods.

**Results:**

1,672,417 advertising PMV were included. 14.6% of advertising PMV were for food and 51.1% of these were for HFSS food. Relative exposure of all viewers to HFSS food advertising increased between study weeks 1 and 2 (odds ratio (99% confidence intervals) = 1·54 (1·51 to 1·57)). Exposure of children to HFSS food advertising did not change between study weeks 1 and 2 (odds ratio (99% confidence intervals) = 1·05 (0·99 to 1·12)). There was almost universal adherence to the restrictions.

**Conclusions:**

Despite good adherence to the restrictions, they did not change relative exposure of children to HFSS advertising and were associated with an increase in relative exposure of all viewers to HFSS advertising. Stronger restrictions targeting a wider range of advertisements are necessary to reduce exposure of children to marketing of less healthful foods.

## Introduction

The prevalence of overweight and obesity has increased rapidly in recent years [1]. Excess bodyweight is associated with an increased risk of a range of non-communicable diseases, including cardiovascular diseases, cancers, chronic respiratory diseases, and diabetes. Consumption of an unhealthy diet is a key contributor to development of overweight and obesity. A number of systematic reviews have concluded that food promotion has an influence on children's food preferences, purchasing requests and consumption [2]–[5]. Although most research focuses on children, who are perceived to be particularly vulnerable to food marketing, there is also evidence that food marketing affects adults' food consumption [6]. As most food marketing focuses on less healthful products [2]–[5], food marketing is likely to play an important role in the development and maintenance of overweight and obesity [7].

Concern over the contribution of food marketing to childhood obesity has led to calls for greater regulation. In 2010, the World Health Organization (WHO) published 12 recommendations on the marketing of food and non-alcoholic beverages to children that were endorsed by the 63^rd^ World Health Assembly [8]. These include a recommendation that member states' “overall policy objective should be to reduce both the exposure of children to, and power of, marketing of foods high in saturated fats, *trans-*fatty acids, free sugars, or salt”(p8) [8].

In response to this growing concern, and preliminary developments leading up to the WHO recommendations, new regulations on the scheduling and content of television food advertising to children in the UK were announced in 2007 [9]. The scheduling restrictions, as fully implemented, prohibit advertisements for foods high in fat, salt and sugar (HFSS) on all children's channels and on non-children's channels during or around programmes ‘of particular appeal to’ 4–15 year olds (see Box S1). The stated aim of the scheduling restrictions was to “reduce significantly the exposure of children under 16 to HFSS advertising” [9].

A number of countries have adopted some form of voluntary or statutory regulation of food advertising to children [10]. In many cases, these tend to focus on ensuring truthful advertising claims, and avoiding the promotion of over-consumption, rather than on exclusion of advertisements for particular foods [10]. Other countries have imposed scheduling restrictions (or total bans) on all advertisements aimed at children, rather than food advertisements in particular (e.g. Norway, Sweden and Quebec, Canada) [10]. However, the UK is the first territory to introduce statutory scheduling restrictions of food advertisements to children [11]. As such, the effects of these restrictions are not known.

We explored the impact of the 2007 UK scheduling restrictions on television food advertising to children on relative exposure to all food advertising and HFSS food advertising among all viewers and among child television viewers, as well as adherence to the restrictions.

## Methods

### Ethics statement

This analysis of anonymised, aggregated data did not include individual human participants and, therefore, did not require ethical review from the UK National Research Ethics Service (see: http://www.nres.npsa.nhs.uk/applications/approval-requirements/ethical-review-requirements/), or the local university research ethics committee (see http://www.ncl.ac.uk/res/research/ethics_governance/ethics/procedures/staff_review.htm).

### Study design

We undertook cross-sectional studies of all advertisements broadcast in the Tyne Tees region of the UK over one week periods before and after the regulations were introduced. The study weeks were the first full weeks of October 2006 (week 1, six months before the introduction of the first phase of restrictions), and July 2009 (week 2, six months after the introduction of the final phase of restrictions). In both cases, broadcast data on what products were advertised were linked to viewing figures data on how many people watched each advertisement. After identification of food advertisements, relevant nutritional data were also added to the dataset and used to calculate the HFSS status of advertised foods.

### Broadcast data

In the UK, a small number of channels have regional variants, whilst most are broadcast nationally. Information on all advertisements broadcast on all channels available in the Tyne Tees region during the study weeks was obtained from an audience research bureau (*Attentional*, Taunton, Somerset, UK). These data included brief information on products advertised, as well as the length, channel and time of broadcast of each advertisement.

### Viewing figures data

*Attentional* also provided viewing data for each advertisement, both for all individuals aged 4 years and older, and separately for children aged 4–15 years. Viewing figures were obtained from an existing UK-wide panel of households selected via a multistage, stratified design to ensure representativeness of all households with televisions across the UK in terms of means of television reception (e.g. terrestrial, cable or satellite), a marker of life stage (pre-family, young family, older family, post family and retired), and social grade. When a household joins the panel, all television equipment in their home is connected to an electronic monitor that determines what is being shown on each device at any one time. All household members register their presence in a room in which a television set is on by pressing the button allocated to them on a handset that accompanies each monitored device.

Viewing data were provided as Television Ratings (TVRs). This is the broadcast industry standard metric for viewing figures and describes the proportion of individuals in the panel who live in a household with equipment to receive each advertisement (i.e. the reference population) that actually watched the advertisement.

The number of individuals in the viewing panel varies between channels broadcast on different platforms (e.g. terrestrial, cable, satellite), between channels with regional variants, and between study weeks. TVRs for channels with regional variants were based on a panel of viewers in the Tyne Tees region (n = 443 in week 1, and n = 496 in week 2). TVRs for channels broadcast nationally were based on panels of viewers across the UK (for terrestrial television: n = 10,913 in week 1, and n = 11,903 in week 2; for other platforms: n = 8,662 in week 1, and n = 11,912 in week 2). The age, gender and socio-economic composition of these panels are described further in Table 1. In each study week, these panels are nested within each other such that all members of the Tyne Tees and national ‘other platform’ panel are also members of the national terrestrial platform panel. There is also some overlap between the Tyne Tees and national ‘other platform’ panels.

**Table 1:** Composition of television viewing panels in 2006 and 2009.

	Tyne Tees regional television		National terrestrial television		National television on other platforms	
	Week 1, n(%)	Week 2, n(%)	Week 1, n(%)	Week 2, n(%)	Week 1, n(%)	Week 2, n(%)
Males	213 (48.1)	240 (48.4)	5,620 (47.2)	5,195 (47.6)	4,205 (48.5)	5,317 (47.5)
Females	230 (51.9)	256 (51.6)	6,283 (52.8)	5,718 (52.4)	4,457 (51.5)	5,875 (52.5)
4–15 years	62 (14.0)	78 (15.7)	1,847 (15.5)	1,781 (16.3)	1,530 (17.7)	1,792 (16.0)
16–24 years	56 (12.6)	55 (11.1)	1,213 (10.2)	1,083 (9.9)	940 (10.9)	1,179 (10.5)
25–34 years	56 (12.6)	55 (11.1)	1,364 (11.5)	1,246 (11.4)	1,099 (12.7)	1,322 (11.8)
35–44 years	62 (14.0)	69 (13.9)	1,802 (15.1)	1,719 (15.8)	1,444 (16.7)	1,728 (15.4)
45–54 years	64 (14.4)	72 (14.5)	1,709 (14.4)	1,482 (13.6)	1,212 (14.0)	1,613 (14.4)
55–64 years	54 (12.2)	82 (16.5)	1,630 (13.7)	1,450 (13.3)	1,112 (12.8)	1,515 (13.5)
65+ years	89 (20.1)	85 (17.1)	2,338 (19.6)	2,152 (19.7)	1,325 (15.3)	2,043 (18.3)
Total	443 (100)	496 (100)	11,903 (100)	10,913 (100)	8,662 (100)	11,192 (100)

### Nutritional data

All advertisements in the broadcast dataset for food and drink (collectively referred to as ‘food’) were identified. We excluded advertisements for alcoholic beverages, food supplements and supermarkets, but included those for restaurants, fast-food chains and fast-food products. Information on the fruit and vegetable and nutritional content of advertised foods was collected and used to determine the HFSS status of each food advertised using the UK Food Standard's Agency's Nutrient Profiling Model [12].

We used a hierarchy of data sources to access nutritional information. Firstly, for advertisements in study week 1, a dataset used in previous research [13], [14] containing nutritional information collected in 2007 on a sample of food advertisements broadcast in 2006 was used where product matches were found. In all other cases, we sourced data during August-September 2009 from, in order of preference: manufacturers' websites and customer care lines, supermarkets' websites and customer care lines, packaging, and standard food table data [15]–[23]. Food table data were used in 3016 of 160,126 (1·9%) of food advertisements.

As the analysis was conducted at the level of the individual advertisement, it was necessary to have a single ‘set’ of nutritional information for each food advertisement. However, in some cases, the information in the broadcast dataset was not detailed enough to identify a single product (e.g. *Branston Relish* listed but six varieties of this product exist). This situation occurred in 77,724 of 160,126 (48·5%) of food advertisements. In these cases, where possible, we imputed nutritional data on the top selling product in the relevant brand range over the four weeks following broadcast of the advertisement (provided by a market research company, *TNS*). Where *TNS* did not have market share data (primarily food sold ‘ready to eat’ by fast-food chains), or a multi-flavour pack was the top seller, the mean nutritional content of all the products in the range or multi-flavour pack, weighted according to relative pack or suggested portion size, was imputed.

### Statistical analysis

To take into account the varying audience size and length of different advertisements, person-minute-views (PMV) for each advertisement were calculated by multiplying the total number of panel members watching any given advertisement (calculated from TVR and reference population count data) by the length of that advertisement, in minutes. To determine the effect of the scheduling restrictions on relative exposure to food advertising and HFSS food advertising, we calculated the number and proportion of all advertising PMV that were for any food and for HFSS food, and the number and proportion of food advertising PMV that were for HFSS foods in each study week. Separate calculations were performed for all viewers and for child viewers. Proportions from week 2 were compared with those from week 1 using odds ratios (OR) and 99% confidence intervals (CI).

To determine if the restrictions were adhered to, we identified all advertisements in study week 2 that were subject to the restrictions as fully implemented (see Box S1). Advertisements on children's channels were identified using a list of all children's channels provided by *Attentional*. Advertisements on non-children's channels that were of particular appeal to 4–15 year olds were identified by first calculating the percentage of panel members watching each advertisement who were 4–15 years old using age-specific TVR and reference population count data. This was then compared to the total proportion of people in the UK population who were 4–15 years old from 2009 population estimates for the UK.

As we were unable to determine exactly what products were shown in all advertisements, we excluded those products where we had imputed nutritional data from market share or weighted means of a range of products from these analyses.

To minimise the risk of type 1 statistical error, a p-value of <0·01 and 99% CI were used to indicate statistical significance throughout. All analyses were conducted using Stata Statistical Software Release 11 (StataCorp. College Station, TX, USA).

## Results

A total of 288 channels broadcast 1,036,953 advertisements over the study weeks. These equated to 1,672,417 PMV of advertising (Table 2).

**Table 2:** Exposure of viewers aged 4 years and older to television food advertising in the UK in 2006 and 2009.

	All advertising	All food advertising		HFSS^1^ food advertising			
Week	PMV^2^	PMV (% of all advertising)	Odds ratio, (99% CI^3^) of advertising PMV being for food	PMV (% of all food advertising)	Odds ratio, (99% CI) of food advertising PMV being HFSS	PMV (% of all advertising)	Odds ratio, (99% CI) of advertising PMV being HFSS
Week 1^4^	704,426	104,145 (14·8)	1·00	40,233 (38.6)	1·00	40,233 (5·7)	1·00
Week 2^5^	967,991	139,959 (14·5)	0·96 (0·95 to 0·98)	84,526 (60.4)	2·19 (2·13 to 2·24)	84,526 (8·7)	1·54 (1·51 to 1·57)
All weeks	1,672,417	244,104 (14·6)	–	124,759 (51·1)	–	124,759 (7·5)	–

1 HFSS = high in fat, salt or sugar;

2 PMV = person-minute views;

3 CI = confidence intervals;

4 first full week of October 2006;

5 first full week of July 2009.

Table 2 shows relative exposure to all food advertising and HFSS food advertising amongst all viewers aged 4 years and older. Overall, 14·6% of advertising PMV were for food. The odds of an advertising PMV being for food was slightly lower in week 2 than in week 1 (OR (99% CI) = 0·96 (0·95 to 0·98)). In total, 51·1% of food advertising PMV and 7·5% of all advertising PMV seen by viewers aged 4 years and older were for HFSS foods. The odds of a food advertising PMV, as well as an advertising PMV, being for HFSS foods was greater in week 2 than in week 1 (OR (99%CI) = 2·19 (2·13 to 2·24) and 1·54 (1·51 to 1·57) respectively). After full implementation of the scheduling restrictions, almost two-thirds (60·4%) of television food advertising seen was for HFSS foods - compared to less than half (38·6%) six months before the regulations were implemented.

Exposure of children aged 4–15 years to all advertising and advertising for food and HFSS food is shown in Table 3. The odds of an advertising PMV seen by children being for food was lower in week 2 than week 1 (OR (99% CI) = 0·85 (0·82 to 0·89)). In contrast, the odds of a food advertising PMV being for HFSS food was higher in week 2 than week 1 (OR (99% CI) = 1·25 (1·15 to 1·37)). Overall, there was no difference in the odds of an advertising PMV seen by children being for HFSS foods in week 2 compared to week 1 (OR (99% CI) = 1·05 (0·99 to 1·12)). After full implementation of the scheduling restrictions, more than half (55·7%) of television food advertising seen by children was for HFSS foods - compared to less than half (43·2%) six months before the regulations were implemented.

**Table 3:** Exposure of viewers aged 4–15 years to television food advertising in the UK in 2006 and 2009.

	All advertising	All food advertising		HFSS^1^ food advertising			
Week	PMV^2^	PMV (% of all advertising)	Odds ratio, (99% CI^3^) of advertising PMV being for food	PMV (% of all food advertising)	Odds ratio, (99% CI) of food advertising PMV being HFSS	PMV (% of all advertising)	Odds ratio, (99% CI) of advertising PMV being HFSS
Week 1^4^	84,264	11,989 (14·2)	1·00	5174 (43.2)	1·00	5174 (6·1)	1·00
Week 2^5^	106,691	13,429 (12·6)	0·85 (0·82 to 0·89)	7476 (55.7)	1·25 (1·15 to 1·37)	7476 (7·0)	1·05 (0·99 to 1·12)
All weeks	190,955	25,418 (13·3)	–	12,650 (49·8)	–	12,650 (6·6)	–

1 HFSS = high in fat, salt or sugar;

2 PMV = person-minute views;

3 CI = confidence intervals;

4 first full week of October 2006;

5 first full week of July 2009.

We assessed adherence to the scheduling restrictions using the 68,545 advertising PMV among child viewers in study week 2 that were subject to the restrictions and where we were able to able to identify the exact food being advertised. Of these, 8 (0·01%) were for an HFSS food product. These 8 PMVs represent one broadcast of one advertisement that had very low viewing figures.

## Discussion

### Statement of principal findings

This is the first detailed evaluation of the effect of the 2007 UK scheduling restrictions on television food advertising to children. In the period between six months before the restrictions began to be implemented and six months after full implementation, we found that exposure of all viewers aged 4 years and older to HFSS food advertising, as a proportion of both all advertising and all food advertising, increased. Exposure of child viewers aged 4–15 years to HFSS food advertising as a proportion of all food advertising increased, but as a proportion of all advertising showed no change. This occurred despite evidence that the scheduling restrictions were widely adhered to.

Whilst effective in excluding HFSS food advertising from the broadcast slots to which they apply, the scheduling restrictions did not achieve the stated aim: “to reduce significantly the exposure of children under 16 to HFSS advertising.” [9] Indeed, they appear to have had a perverse effect of increasing exposure of all viewers to HFSS food advertising.

### Strengths and weakness of the study

We relied on secondary data throughout and this imposed a number of limitations. With more than one million advertisements over 288 channels, it was impossible to view all of the advertisements included in the analysis. This made it difficult, in many cases, to identify exactly what food products were being advertised. Estimating nutritional content using market share data or weighted mean nutritional content from a range of products was systematic and objective, but not ideal. For this reason, when exploring whether or not the restrictions were adhered to, we excluded those food advertisements for which we had used market share or weighted mean data to attribute nutritional information. Our assessment of adherence to the scheduling restrictions may, therefore, be conservative.

The broadcast data we used were supplied by an audience research bureau. Given the reliance of the advertising, manufacturing and retail industry on such data, they are likely to be the most accurate available. However, we do not have any information on how accurate these data are and it is possible that they are subject to some reporting bias. Furthermore, as we were only able to access group-level, rather than individual-level, data we were unable to determine the effects of the regulations on individual exposure to HFSS advertisements. Nor were we able to study socio-economc differences in exposure or the impact of the restrictions on either purchasing, consumption or bodyweight.

As far as possible we used manufacturers' data on the nutritional content of advertised foods. This represents the most product specific information available. However, the majority of nutritional information was collected in 2009. As foods are constantly being reformulated, with recent emphasis on reducing salt, fat and sugar content [24]–[26], our approach may underestimate the proportion of foods advertised in week 1 that were HFSS. Furthermore, as we were unable to determine the specific product being advertised in almost 50% of food advertisements, we had to impute estimated data from a number of sources. Whilst this imputation may have reduced the accuracy of our results, we do not believe that it introduced any systematic bias in favour of either more or less HFSS foods.

Given the observational nature of our data, we cannot conclude that changes seen in advertising were necessarily a result of the new scheduling restrictions. Other, contextual, factors may also play a role. For example, we only studied two individual weeks and these may have been influenced by particular advertising campaigns or seasonal differences in advertising between October (week 1) and July (week 2). Finally, our data only chart short term changes following the introduction of the restrictions. Further work is required to determine the longer terms effects and to confirm that our results are not particular to the individual weeks studied.

### Strengths and weaknesses in relation to other studies

Our use of PMVs takes into account that different advertisements have different lengths and are watched by different numbers of individuals and so reflects exposure better than the simple advertisement count methods used in previous analyses of UK television food advertising [13], [14]. Further, our inclusion of all UK commercial channels provides a much more comprehensive picture than previously reported [13], [14].

We believe our evaluation also represents a significant improvement on interim and final reviews of the new scheduling restrictions commissioned by OfCom, the UK communications regulator [27], [28]. In particular, these reviews used substantially different methods to determine the HFSS status of food advertisements broadcast before and after implementation of the restrictions, neither of which were based on product-specific nutritional data, as used here.

### Interpretation of findings

It has previously been highlighted that little research exists on the effect of different approaches to regulating food marketing to children [10]. As identified above, evaluations of the UK scheduling restrictions commissioned by OfCom suffered from substantial methodological limitations meaning their results are unlikely to be valid. Other strategies for regulating food marketing to children include self-regulation, whereby the food industry itself defines how it will behave and how this will be regulated. This is an increasing global phenomenon [29] but it has been convincingly argued that such self-regulation is unlikely to ever lead to wholesale change in the balance of what food is marketed to children [30].

Our finding that exposure of children to HFSS food advertising, as a proportion of all advertising seen, did not change despite good adherence to the restrictions reflects the fact that children watch a wider range of television than just those programmes particularly targeted at them. By focusing on only a subset of all advertisements that children are exposed to, the UK scheduling restrictions appear to have been flawed from the outset. Future policies should consider including a much wider range of advertising - for instance, by using a time-based ‘watershed’, as proposed by recent guidance from the UK National Institute for Health and Clinical Excellence [31].

Perversely, we found that exposure to HFSS food advertising, as a proportion of both all advertising and all food advertising seen, increased among all viewers following introduction of the scheduling restrictions. This indicates that rather than reducing HFSS advertising, advertisers may simply have responded to the scheduling restrictions, at least in part, by moving when and on what channels HFSS advertisements were broadcast. It is also possible that some marketing of HFSS foods was moved from television to much less regulated spheres, including on-line. The indication of a shift in scheduling of HFSS advertising is particularly concerning in light of recent evidence that exposure to food advertising effects the food consumption of adults, as well as children [6]. Food marketing of less healthful foods should be considered a threat to whole population health, and not just that of children. All aspects of marketing, and not just television advertising, should also be considered when developing regulatory policy.

Our evidence of good adherence to the restrictions suggest that scheduling restrictions on television advertising of HFSS foods can be effective in reducing broadcast of these advertisements – but only in the broadcast slots to which they apply. Without wide ranging consideration of all broadcasting, such regulations run the risk of simply shifting, rather than reducing or eliminating, the problem.

### Conclusion

Despite evidence of good adherence to the new scheduling restrictions on television food advertising to children, we found that exposure of children to advertisements for ‘less healthy’ foods was unchanged following their introduction. Exposure of all viewers to advertisements for ‘less healthy’ foods increased following introduction of the restrictions. The restrictions did not achieve their aim and this is likely to be because they only applied to a very small proportion of all television broadcast. Further interventions will be needed to achieve a reduction in exposure of children to ‘less healthy’ food advertising.

## Supporting Information

Box S1 **Details of the 2007 UK scheduling restrictions on television food advertising to children** [**9**] **.**
(DOCX)
