# Supplementary material for: Effect of Restrictions on Television Food Advertising to Children on Exposure to Advertisements for ‘Less Healthy’ Foods: Repeat Cross-Sectional Study
Source: PLoS One. 2012 Feb 15;7(2):e31578. doi: 10.1371/journal.pone.0031578 (PMC3280312; doi:10.1371/journal.pone.0031578)
Supplement: Box S1 — Details of the 2007 UK scheduling restrictions on television food advertising to children [9] . (DOCX) [file pone.0031578.s001.docx]

**Box S1 – Details of the 2007 UK scheduling restrictions on television food advertising to children[**[**9**](#_ENREF_9)**]**

*Applicable foods*

The restrictions apply to foods prominently displayed in advertisements that are high in fat, salt and sugar (HFSS). HFSS foods are those identified as ‘less healthy’ by the UK Food Standards’ Agency’s Nutrient Profiling Model.[[12](#_ENREF_12)] This model allocates a score to food and non-alcoholic drinks, based on the balance of ‘negative’ constituents (energy, saturated fat, sugar, and sodium) to ‘positive’ constituents (fruit, vegetables and nuts; fibre; and protein).

Advertisements that refer to, but do not show, foods are not covered by the restrictions, whether or not they are HFSS. This includes advertisements for food brands and ranges, even if they are strongly associated with HFSS products.

Foods that are shown incidentally, for instance in the background of a general kitchen scene, are not covered by the restrictions, whether or not they are HFSS.

*Applicable broadcast slots (after full implementation)*

All advertising slots on children’s channels.

Advertising slots on non-children’s channels immediately before, during or after programmes “of particular appeal to” children – when the proportion of people watching who are children is more than 120% of the proportion of people in the UK population who are children.

*Implementation timetable*

From April 2007 – monthly minutage devoted to HFSS advertisements on children’s channels to be not more than 75% of average monthly minutage devoted to HFSS advertisements on that channel in 2005. No advertising of HFSS products before, during or after programmes of particular appeal to children aged 4-9 years on non-children’s channels.

From January 2008 – monthly minutage devoted to HFSS advertisements on children’s channels to be not more than 50% of average monthly minutage devoted to HFSS advertisements on that channel in 2005. No advertising of HFSS products before, during or after programmes of particular appeal to children aged 4-15 years on non-children’s channels.

From January 2009 – full implementation. No HFSS advertisements on children’s channels. No HFSS advertising on non-children’s channels before, during or after programmes of particular appeal to children aged 4-15 years.
